# Light-Activated ZnO Sensor for NO_2_ Detection at Room Temperature

**DOI:** 10.1021/acsomega.6c04616

**Published:** 2026-10-03

**Authors:** Ailyn Estévez, Joana Pimenta, Andoni Beriain, Noemí Pérez

**Affiliations:** † Tecnun-School of Engineering, 54393 University of Navarra, 20018 San Sebastián, Spain; ‡ CeNTI-Center of Nanotechnology and Smart Materials, 4760-034 Vila Nova de Famalicão, Portugal

## Abstract

Nitrogen dioxide (NO_2_) is a pollutant gas that causes deterioration of the air quality. This work provides a promising approach to efficiently detecting NO_2_ at room temperature using a chemiresistive sensor based on a zinc oxide (ZnO) ink on top of an interdigitated electrode (IDE). We propose a high-performance and low-cost gas sensor composed of aluminum electrodes printed on a flexible substrate using thermal printing. Unlike other gas sensors, the ZnO-based sensor works under ambient conditions and artificial light from fluorescent lamps, with no need for a dedicated light source. Changes in the sensor resistance are measured for low concentrations of NO_2_ gas adsorbed on the ZnO surface, showing a linear increase in resistance when the concentration of NO_2_ increases from 0.1 to 1 ppm. The advantages of this sensor are high sensitivity, fast response time, full recovery, good selectivity, stability over time, low power consumption and low cost.

## Graphical abstract

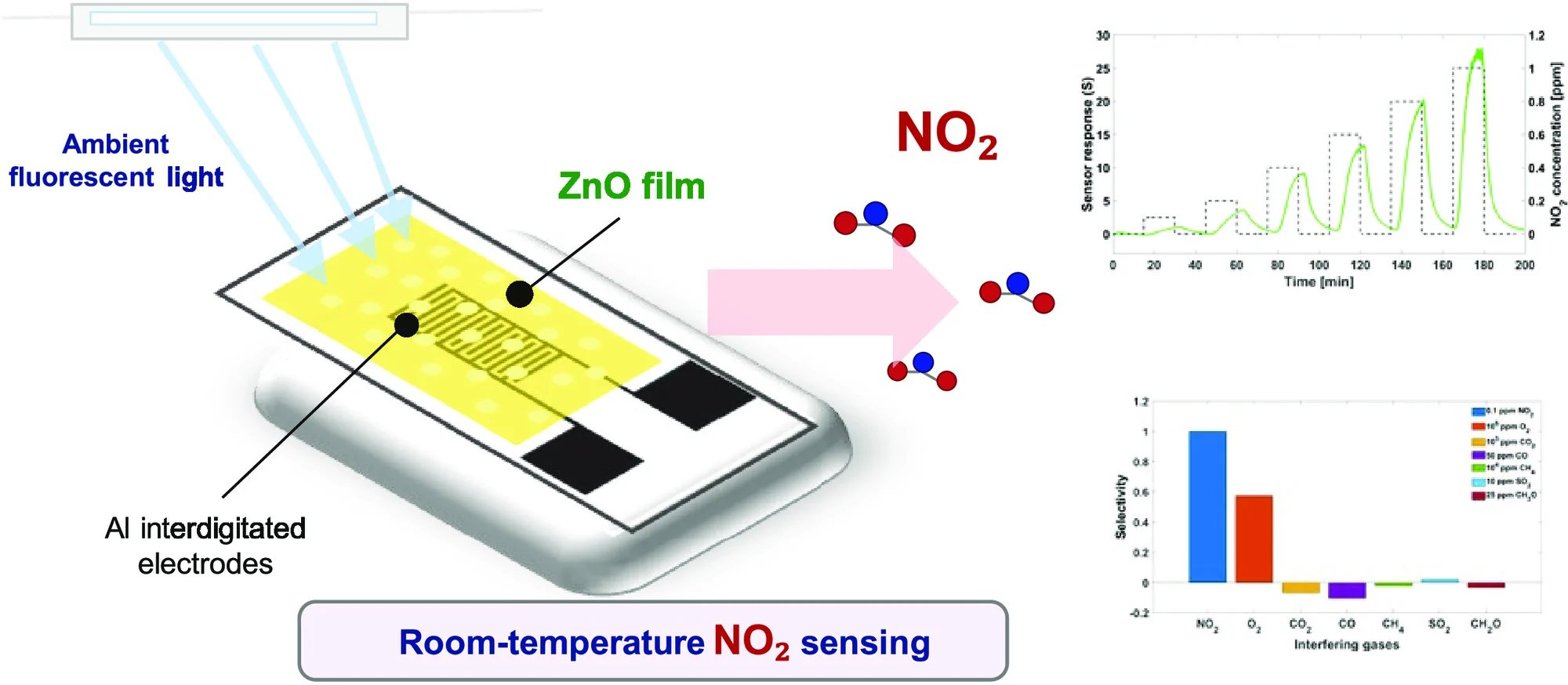

## 1 Introduction

Nitrogen dioxide (NO_2_) is one of the most harmful air pollutants derived from industrial combustion and automobiles, which affects human health at parts per billion (ppb) and parts per million (ppm) levels.^1^ Prolonged exposure to NO_2_ can cause lung tissue inflammation and some respiratory diseases, such as bronchitis, pulmonary edema and asthma.^2,3^ This pollutant gas contributes negatively to the environment, causing acid rain and photochemical smog. Therefore, high-performance sensors are needed for detecting and monitoring parts per billion (ppb) and parts per million (ppm) levels of NO_2_ in the atmosphere. In the last decade, several NO_2_ sensors have been developed, such as piezoelectric, electrochemical and semiconductor sensors.

Nowadays, semiconductor gas sensors are becoming more attractive in NO_2_ detection because of their low cost, simple structure and long-term stability. These sensors are frequently based on metal oxides such as ZnO, WO_3_, NiO, In_2_O_3_, TiO_2_, or some composite materials that exhibit good performance in NO_2_ detection.^4–6^ However, these gas sensors often operate at high temperatures, leading to undesirable long-term power consumption.^7^ Some researchers have reported feasible approaches to lower the operating temperature, such as doping with noble metals,^8,9^ construction of heterojunction structures,^10–12^ hybrid composites with conducting polymers,^13^ surface oxygen vacancies adjustment,^14–17^ and light-assisted stimulation with visible^9^ or ultraviolet (UV) light.^10,14,18^ These strategies can not only lower the operating temperature but also improve the sensor performance and its reversibility.

When designing gas sensors for room-temperature operation, practical implementation requires consideration of sensitivity, speed (response and recovery rate), and selectivity of the sensor. However, many of the metal oxide-based gas sensors reported for room-temperature operation suffer from poor selectivity since they respond to various gases with the same sensing mechanism, such as, for example, the surface depletion model. In this sensing mechanism, the resistance of the sensor is dependent on the charge transfer between the metal oxide and the surface-adsorbed gaseous species.^9,11,14,16,19^ Several strategies have been developed to improve the selectivity of sensors for a particular gas. For instance, surface functionalization of metal oxides with new metal dopants or nanoparticles has been widely used to improve selectivity toward reducing gases.^20^ However, doping with a metal decreases the sensitivity of the material because of the lower concentration of the functional molecules. Other alternatives for improving the selectivity of the gas sensor include modifying the electronic properties of metal oxides through the creation of heterojunctions,^11,18^ coating metal oxide nanoparticles with molecular sieves,^21^ and the recent development of gas selectivity control through light illumination.^8^

As mentioned before, many sensors working at room temperature face problems of poor reversibility and low sensitivity. The limited surface activity of metal oxides at room temperature poses a challenge to the adsorption-desorption and electron-transfer processes. As a result, metal oxides typically exhibit insufficient and irreversible responses to NO_2_ at room temperature, especially in the dark.^14^ Light, with wavelengths ranging from UV to visible, has been widely employed to improve the sensitivity and reversibility as well as the response rate of metal oxide-based gas sensors working at room temperature.^9,10,14,18^ However, in the case of light-assisted sensing, the need for a dedicated light source, typically a visible or UV LED, increases the cost and the power consumption of the device. The inclusion of metals and nonmetals in the semiconductor is a method that allows the displacement of its absorption threshold, normally from the UV region toward the visible.^9,22^ The challenge lies in the fact that to enhance sensor sensitivity and response rate, specific light conditions (such as wavelength and intensity) are typically required. Attending to sensor recovery, some researchers use vacuum environments,^23^ although the influence of light is considered a more viable strategy for real applications. In particular, UV stimulation can promote the desorption process and it is typically used in the sensor recovery stage to accelerate and achieve complete recovery. In this work, we explore the use of ambient light from fluorescent lamps in the laboratory to achieve high performance of the gas sensor at room temperature. Moreover, we studied the sensor response using different intensities of UV and visible stimulation for a complete understanding of the light-assisted method.

Metallic interdigitated electrodes (IDEs) made of gold, silver, or platinum are commonly used in the development of gas sensors. Often, these IDEs are used, increasing the cost of the sensor. With the aim of developing a low-cost sensor, we propose the use of aluminum, which allows quick, cheap, and easy production of IDEs on a flexible substrate by thermal printing. On top, we applied a ZnO ink and evaluated the sensing characteristics of the sensor based on this metal oxide during its operation under different lighting conditions.

## 2 Materials and Methods

### 2.1 Detection Mechanism

Chemoresistive gas sensors operate on the principle that the electrical resistance of a sensing material is controlled by the surrounding atmosphere. The introduction of a target gas produces a change in the resistance/conductivity of the sensitive material, in this case, zinc oxide (ZnO), compared to that obtained with a stable background gas (e.g., dry air). The response of the resistive gas sensor is typically described in terms of two functions, called the receptor and transducer principles.^16^ These principles describe the chemical interaction between the material (metal oxide) and the target gas and how this interaction is transduced into an electrical signal.

Zinc oxide (ZnO) is an n-type semiconductor whose electrical and surface properties are strongly influenced by intrinsic defects, particularly oxygen vacancies, which can act as electron-donating defects.^23^ Beyond their contribution to the electronic properties of ZnO, oxygen vacancies can also provide active sites for the adsorption and activation of the gas molecules. Thus, the sensing process is governed not only by the electron density of the semiconductor but also by the concentration and chemical state of surface oxygen vacancies, which influence the adsorption of oxygen and the subsequent interaction with the target gas.

In the presence of molecular oxygen, which acts as an electron acceptor, electrons from the conduction band of ZnO are transferred to adsorbed oxygen species. This results in the chemisorption of the oxygen molecules on the surface of the material and at the grain boundaries, forming O_2_^–^, O^–^, or O^2–^, depending on the temperature.^23,24^

Since the experiments in this work will be carried out at room temperature (<100 °C), the process can be described by the following reaction

1 $$O_{2}\left(ads\right)+e^{-}\to O_{2}^{-}$$

where O_2_(ads) denotes molecular oxygen adsorbed at the ZnO surface, and *e* ^–^ represents a conduction electron of the semiconductor.

Oxygen vacancies can facilitate this process by providing favorable surface sites for oxygen adsorption and modifying the local electronic structure of the ZnO surface. Consequently, the oxygen vacancy concentration can influence both the amount of chemisorbed oxygen and the associated surface charge transfer. This defect-mediated contribution is particularly relevant under illumination, where the surface oxygen vacancy population can be dynamically modified, leading to enhanced room-temperature NO_2_ sensing.^12^

As oxygen anions extract electrons from the ZnO, as depicted in Figure 1a, an electron depletion layer is formed near the surface. In the polycrystalline sensing film, this surface depletion modifies the potential barriers at the grain boundaries, resulting in an increase in the resistance of the material. When the semiconductor is exposed to light, photogenerated carriers modify the surface charge state and can enhance the interaction between the ZnO surface and the adsorbed oxygen. Therefore, the establishment of the baseline under illumination can be understood as the result of the coupled equilibrium among oxygen adsorption/desorption, charge transfer and the dynamic evolution of surface oxygen vacancies.

**Figure 1:**
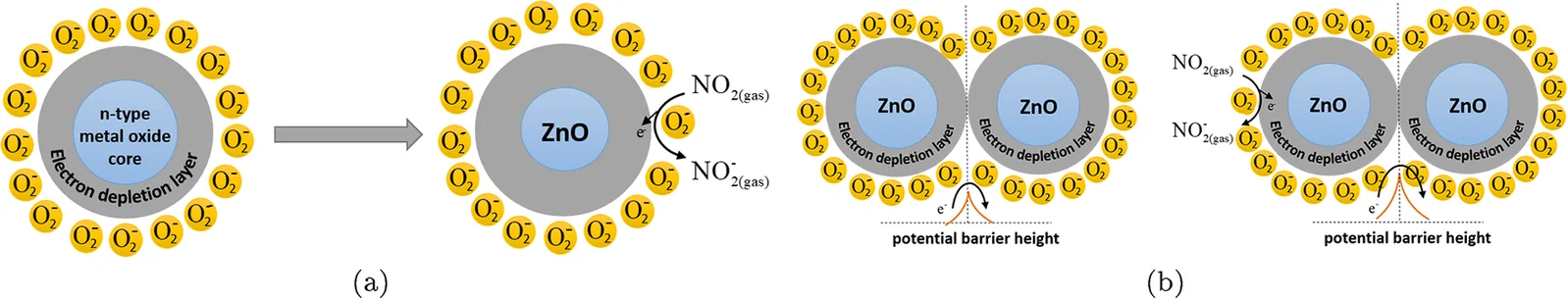
Schematic illustration of ZnO n-type semiconductor material (a) exposed to the background atmosphere and an oxidizing gas, (b) showing the potential barrier height change at the grain boundaries.

In the presence of NO_2_, a charge-carrier trapping effect takes place because NO_2_ is a strongly oxidizing gas that withdraws electrons from the n-type ZnO. The interaction of NO_2_ with the ZnO surface can occur at defect sites, including oxygen vacancies, and can also involve preadsorbed oxygen species. This interaction results in additional electron withdrawal from the semiconductor, producing a wider depletion layer and increasing the potential barrier at the grain boundaries, as illustrated in Figure 1b. Oxygen vacancies act as active surface sites that facilitate the NO_2_ adsorption and interfacial charge transfer. Recent in situ measurements demonstrate that oxygen vacancy concentration decreases during NO_2_ exposure and recovers dynamically when the material is returned to air.^12^

Moreover, illumination promotes the surface reactions associated with NO_2_ sensing by modifying both the charge-carrier population and the concentration/state of oxygen-vacancy sites. In this context, the enhanced response under illumination cannot be attributed solely to the generation of electron–hole pairs; rather, it can be understood as the combined effect of photo-induced charge-carrier dynamics and oxygen-vacancy-mediated surface reactions.

When the target gas is removed and the sensor is exposed again to air, the recovery of its baseline resistance takes place gradually. This recovery involves the desorption and/or transformation of the adsorbed NO_2_^–^ derived surface species, together with the re-establishment of oxygen adsorption. This desorption process takes advantage of the applied light, which promotes the desorption of NO_2_^–^.^9,25^ As the surface returns toward its initial state and the vacancy population re-equilibrates, electrons are restored to the conduction pathways of ZnO, the depletion layer becomes thinner, and the grain-boundary potential barriers decrease, returning the sensor resistance toward its baseline value.

### 2.2 Gas Sensor Fabrication and Characterization

Zinc oxide (ZnO) is an n-type semiconductor with a wide band gap (*E*_*g*_ ≃ 3.37 eV) at room temperature.^26^ Due to its excellent chemical stability and electrical properties, it has become the most widely used sensitive material for NO_2_ detection.^24^ In this work, the aim is to develop a low-cost and high-sensitivity sensor at room temperature. For this purpose, a ZnO ink was prepared by mixing 30 g of a ZnO dispersion in water (a commercial ink of 20 wt % in water purchased from Sigma-Aldrich), 5 g of a commercial water-based (WB) emulsion of 30 wt % solids in water, and 15 g of deionized water.

The substrate used for the fabrication of the gas sensor (Thermalfilm Select@21940) was a 51 μm thick polyester film. The aluminum interdigitated electrodes with a finger and gap width of 3 mm were patterned by thermal printing using a standard ZT411 Zebra printer. The aluminum conductive ribbon (σ = 6.2 × 10^5^ S/m) has a thickness (*h*_*t*_) of 4.5 μm.

The prepared ZnO ink was drop-cast on top of the interdigitated electrodes. A vinyl mask was used to define the coated area, exposing an active sensing area of 15 mm × 10 mm (see Figure 2). The deposited volume, 20 μL, was selected as optimal in order to obtain a homogeneous layer that covered the sensing area without visible agglomerations. After deposition, the samples were dried at room temperature (RT) for 24 h. Approximately a 6 μm thick zinc oxide (ZnO) semiconductor film was obtained after that process, as determined from 3D optical profilometry measurements performed at different positions of the deposited layer. The resulting film showed resistance values around 20 MΩ when illuminated with the fluorescent lamps of the laboratory. As for the resistance of the film in darkness, it varied around hundredths of MΩ, the mean value being 485 M*Ω*.

**Figure 2:**
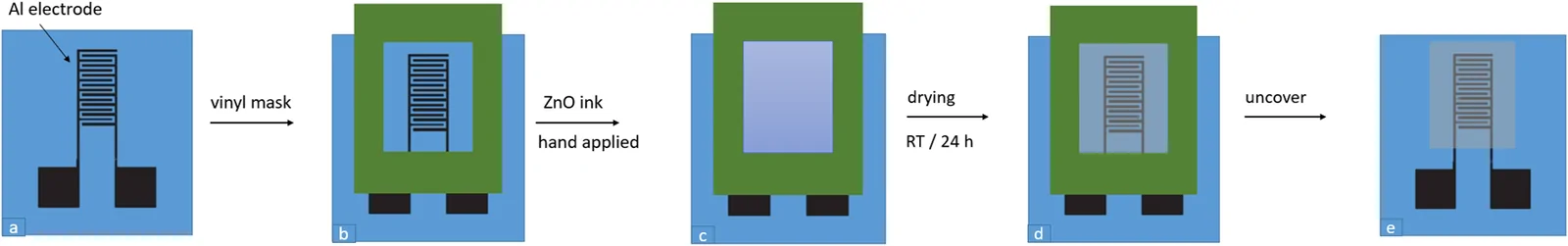
Schematic illustration of ZnO sensing film deposition process: (a) printed aluminum interdigital electrodes; (b) vinyl mask placed on top of the electrodes; (c) applied ZnO ink; (d) ZnO ink after drying; and (e) final ZnO sensor.

The sensing properties of the ZnO chemoresistive sensor to NO_2_ were evaluated at room temperature with and without illumination. Figure 3 shows the experimental setup used for that purpose. The ZnO-based sensor is inside the chamber and connected to a sourcemeter (Keithley 2450) for the continuous evaluation of the electrical resistance. The chamber was sealed using a transparent material that allowed light from fluorescent lamps to pass through.

**Figure 3:**
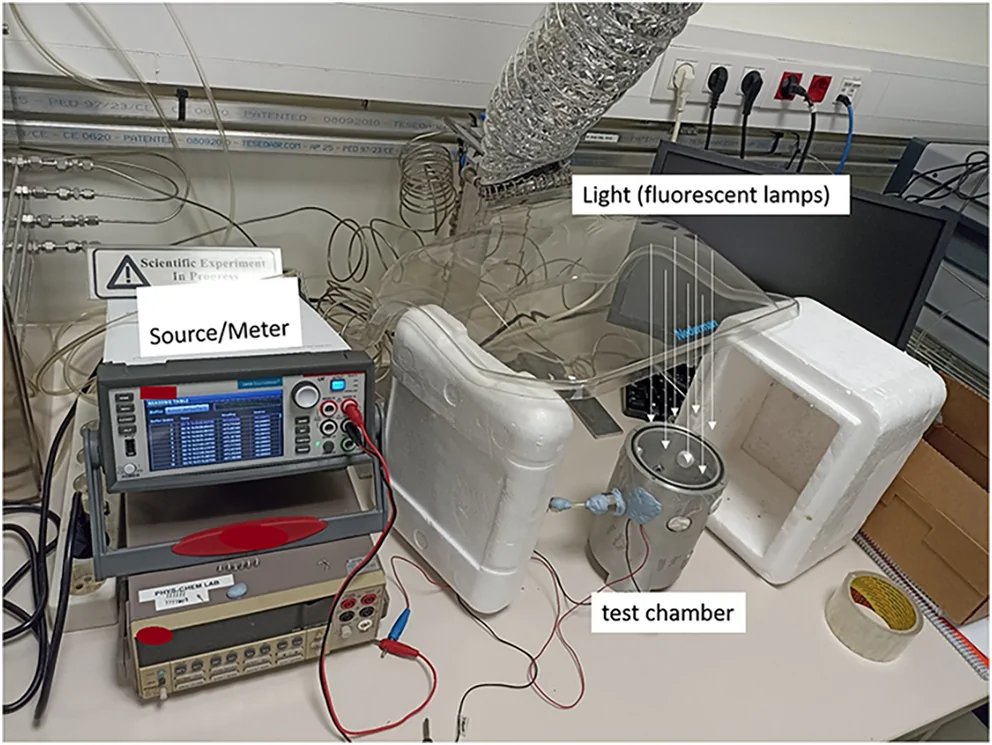
Experimental setup for ZnO gas sensor characterization under light stimulation at room temperature.

The gas sensor was characterized by its sensor response (*S*) defined as (*R*_*g*_–*R*_*a*_)/*R*_*a*_, where *R*_*a*_ and *R*_*g*_ are the resistances of the sensor when exposed to air and NO_2_, respectively. Other parameters such as response time, recovery time, selectivity and stability under light stimulation at room temperature were also measured. In addition, the influence of the post-annealing treatment on the response of the sensor was analyzed. To characterize their sensing performance, the fabricated sensors were placed in a gas chamber with an inlet and an outlet. The NO_2_ original gas comes from a 50 ppm bottle mixed with dry air. The input concentration of NO_2_ was varied from 0.1 to 1 ppm by mixing the 50 ppm original NO_2_ with dry synthetic air with the help of a Bronkhorst mass flow controller in an automatic LabVIEW-controlled setup. The flow rate of the mixing gas was 400 sccm (standard cubic centimeters per minute). The gas feeding time of each cycle was fixed at 15 min for all tests. The resistances of the films were measured using the sourcemeter (Keithley 2450) at room temperature (20.5 °C).

In order to evaluate the influence of light on the response of the sensor, it was exposed to UV and white light from LEDs. The light intensity from the different light sources was characterized with a power meter (New Focus 3801) and a light sensor (New Focus 3822) at different wavelengths (400 and 532 nm).

## 3 Results

### 3.1 Sensor Response to NO_2_ Detection

The response (*S*) of the ZnO-based sensor upon exposure to NO_2_ concentrations ranging from 0.1 to 1 ppm under ambient light at room temperature is shown in Figure 4a, and the linear fit of the corresponding response versus NO_2_ concentration is plotted in Figure 4b.

**Figure 4:**
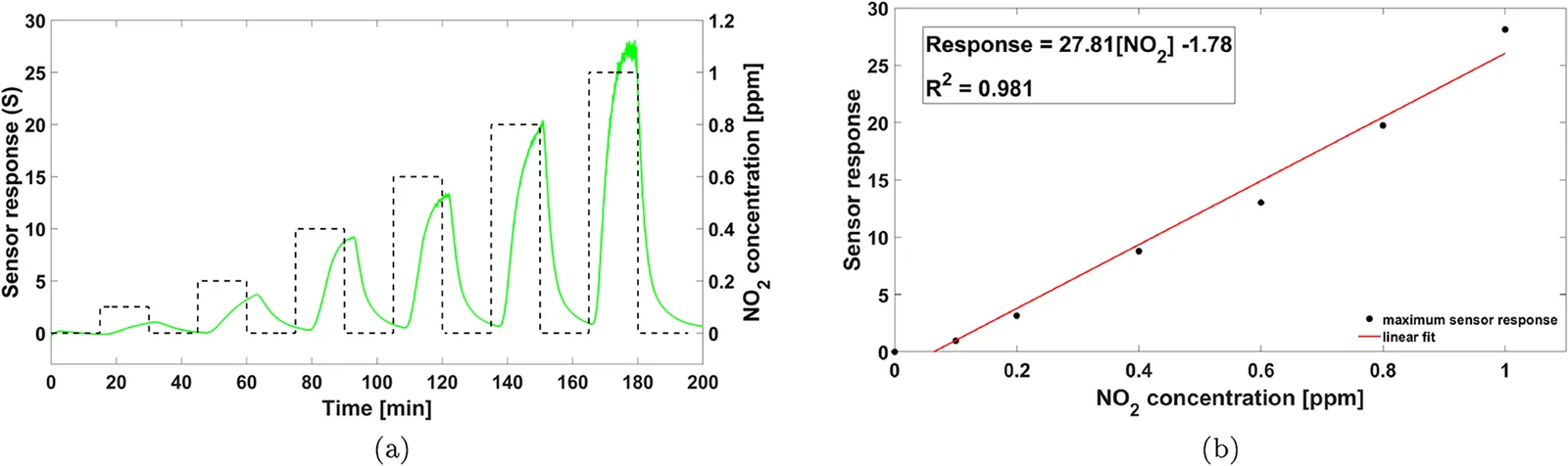
(a) Response of the ZnO-based sensor to different concentrations ranging from 0.1 to 1 ppm of NO_2_ at room temperature under fluorescent illumination. (b) Calibration curve based on a linear fit (slope = 27.8) of the sensor responses as a function of NO_2_ concentration.

Upon exposure to NO_2_, the resistance of the sensor increases as expected since NO_2_ is an oxidizing gas, and the sensor response increases with increasing NO_2_ concentration. The sensitivity of the gas sensor is determined as the slope of the calibration curve, which is constructed by using the maximum response of the sensor at the concentrations tested.

It can be seen that the resistance increases and reaches saturated values when the NO_2_ is injected. Moreover, when NO_2_ is gradually replaced by dry air within 15 min, the resistance decreases rapidly to near the baseline. Figure 4b shows that there is good linearity between the sensitivity response of the gas sensor and the increasing concentration of NO_2_.

The resistance of the gas sensor increases or decreases depending on the adsorption or desorption of NO_2_ at the grain boundaries, as explained in Section 2.1. The electrical resistance of the sensing device is determined by the height of the potential barrier at the grain boundaries, which is related to the depletion width and the effective number of electrons captured by surface acceptors. When NO_2_ is introduced into the chamber with the ZnO-based sensor, the surface depletion layer is enlarged, resulting in an increase in electrical resistance due to the adsorption of NO_2_. The recovery of the sensor is achieved under light illumination. When the sensor is no longer exposed to the target gas and is instead exposed to air, the desorption of NO_2_^–^ and oxygen ions depends on the photon energy received from the light. O_2_^–^ and NO_2_^–^ ions adsorbed at the grain boundaries of the ZnO film receive the necessary energy to release the electrons back to the ZnO core, thus reducing the width of the electron depletion layer, which increases the conductivity of the ZnO layer and lowers the resistance to the baseline.

During this study, the influence of artificial light on the sensor response was evident. Therefore, we analyzed in detail the performance of the sensor under different lighting conditions. First of all, we compared the response of the sensor under the illumination of the fluorescent lamps in the laboratory and in the dark. Figure 5 illustrates the response of the sensor for two cycles of 0.1 ppm of NO_2_ and dry air at room temperature, one cycle under fluorescent light and the other in darkness. The sensor can detect the chemical changes in the surrounding atmosphere in darkness reaching extremely high resistance values (beyond the measurement range of the sourcemeter greater than 1 GΩ). However, it requires exposure to light to return to its baseline state, as expected.

**Figure 5:**
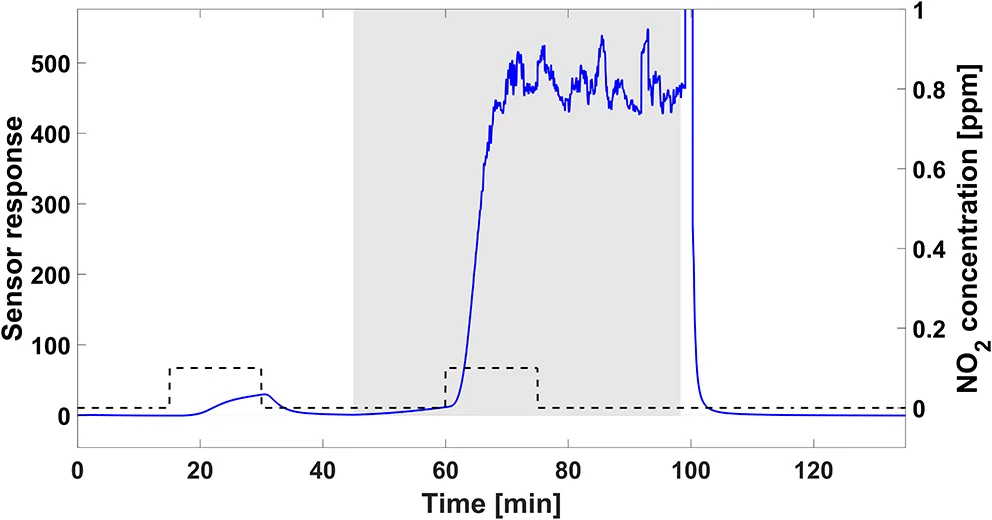
Two cycles of 0.1 ppm of NO_2_ and dry air at room temperature, one cycle under fluorescent light and the other in darkness (marked in gray).

In a second experiment, the sensor was exposed to the illumination of two LEDs (UV and white). In the experimental setup, the gas chamber was closed with the LED inside attached to the lid at a distance of 8 cm from the sensor.

To evaluate the effect of light intensity on the sensor response, the ZnO sensor was exposed to the same N*O*_2_ concentration pulse of 1 ppm while varying only the LED illumination intensity. Figure 6a shows the response under UV LED illumination, with optical intensities of 0.28, 13, and 75 μ W cm^–2^ measured at 400 nm. Figure 6b shows the response under white LED illumination, with optical intensities of 310, 400, and 485 μ W cm^–2^ measured using 532 nm as the reference wavelength. In both cases, the dashed black line represents the applied NO_2_ concentration profile. The resistance axis is shown on a logarithmic scale to facilitate comparison between the different responses.

**Figure 6:**
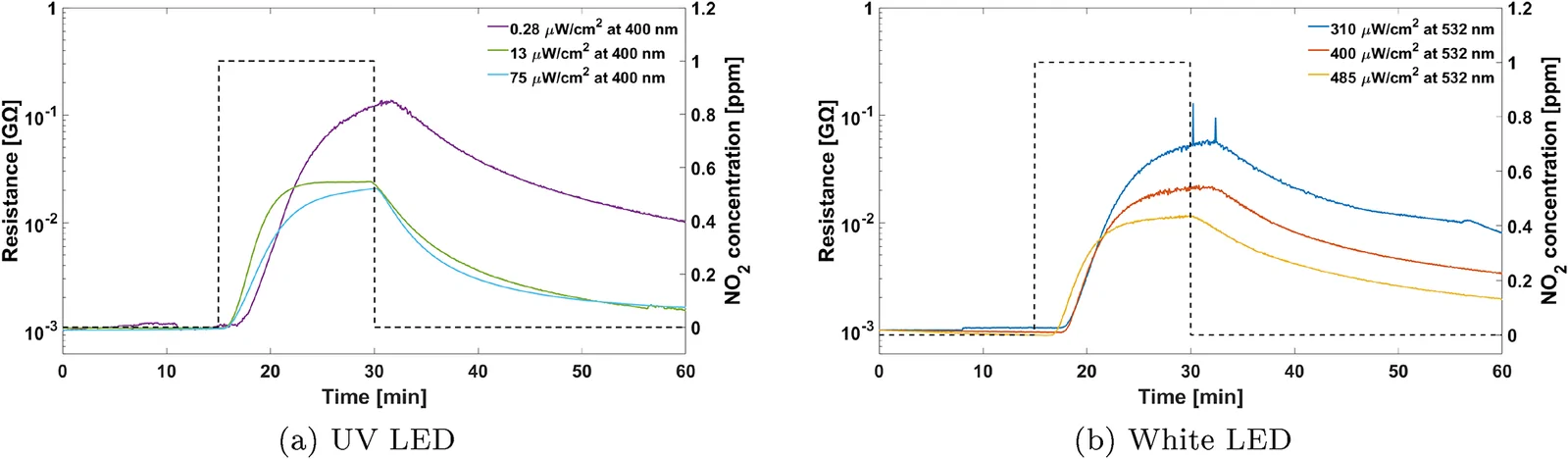
Resistance variation of the ZnO-based gas sensor under different LED illumination intensities during exposure to 1 ppm of NO_2_: (a) UV LED intensity measured at λ = 400 nm and (b) white LED intensity measured at λ = 532 nm.

Figure 6 clearly shows that as light intensity increases, the sensor response decreases for both UV (a) and white (b) light. Moreover, poorer sensor recovery is observed mainly with visible light. It is clear from these results that enough UV light intensity is needed for the photo-induced desorption mechanism to take place, in order to promote the desorption of NO_2_^–^ ions at the ZnO surface.

In the case of fluorescent light, an intensity of 21 μ W cm^–2^ is obtained at a wavelength of 400 nm, and 15 μ W cm^–2^ is obtained at a wavelength of 532 nm. This result shows that the intensity of the UV light coming from the fluorescent lamp is enough to provide the recovery of the baseline of the sensor. As the content of UV light is greater in daylight than in fluorescent lamps, this sensor would work perfectly in daylight conditions. However, the variability of natural light has to be taken into account in the performance of the sensor.

The response and recovery times of the ZnO-based sensor toward various concentrations of NO_2_ under UV and white light are also evaluated. The response time is defined as the time required until 90% of the whole response is reached, while the recovery time denotes the time needed until 90% of the signal is recovered.^27,28^ The data points in Figure 7 correspond to response and recovery times extracted from individual measurements performed under different illumination conditions. Each marker represents the value obtained for a specific combination of NO_2_ concentration, LED type, and illumination intensity, as indicated in the legend.

**Figure 7:**
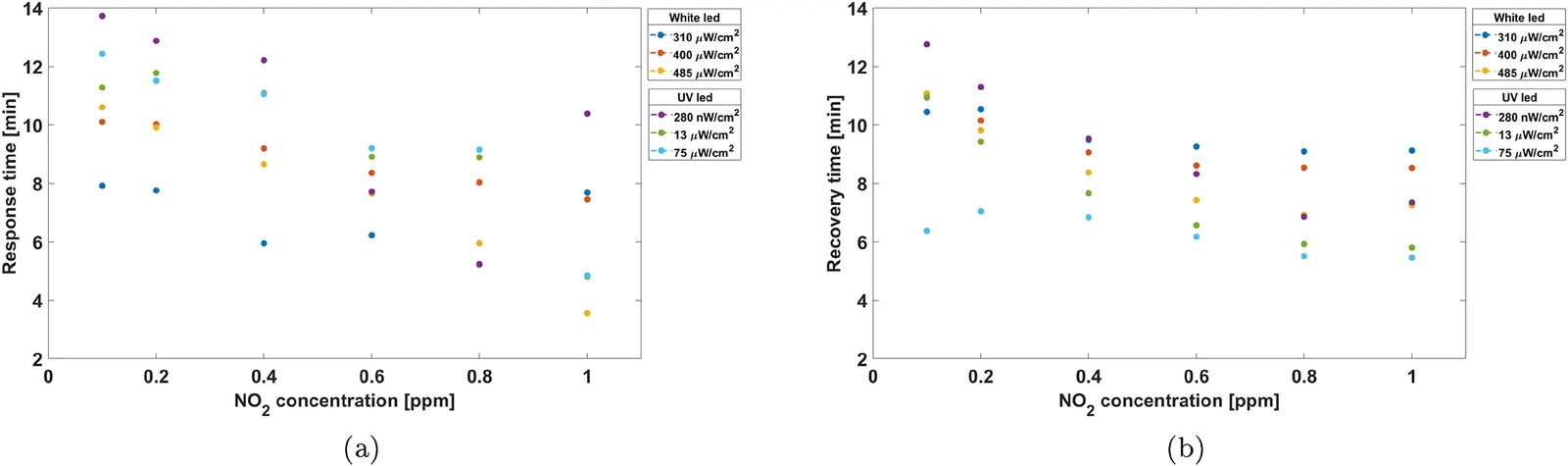
(a) Recovery and (b) response times of ZnO-based gas sensor for NO_2_ detection under white and UV LEDs illumination at room temperature. Each marker corresponds to a specific illumination condition and NO_2_ concentration.

As can be seen in Figure 7(a), the response time shows a decreasing trend with gas concentration. This tendency is maintained for different illumination intensities. Moreover, the response time increases with an increase in light intensity. Although a shorter response time would be expected for higher target gas concentrations, this is not the case for 1 ppm under lower light intensity for either white or UV LEDs. The light energy promotes the movement of the photogenerated electrons, intensifying the detection and, even more, the recovery of the sensor. However, for a concentration of 1 ppm, the photon energy seems insufficient to speed up the gas detection process for the chemiresistive sensor under a lower light intensity.

In addition, the recovery time is reduced with decreasing wavelength of light when comparing white light with UV light, which is consistent with other studies.^9^ The explanation is based on the photon energy (*E* = *hc*/λ), which increases with decreasing light wavelength. As increasing photon energy enhances the desorption of NO_2_^–^ species, which increases the surface-accumulated electrons of the sensing material, the baseline resistance declines faster.

Environmental humidity is a critical factor that can affect the sensing performance of ZnO-based sensors. To evaluate the effect of humidity on the sensor response, an experiment is conducted in which the relative humidity (RH) is varied. The NO_2_ concentration is maintained fixed at 1 ppm, and this gas is mixed with humid air in a collector with relative humidity levels of 25%, 50%, and 75%, set and measured with an automatic control system. As can be seen in Figure 8, the sensor response exhibits a nonmonotonic dependence on relative humidity.^29^ The response increases from 0 to 50% RH, whereas a further increase to 75% RH results in a decrease in the sensor response. At low or moderate relative humidity, water molecules chemisorb and dissociate on the ZnO surface, forming hydroxyl groups (OH^–^) at oxygen defect sites, which act as active centers that lower the charge-transfer barrier and enhance NO_2_ adsorption.^5,30^ However, at higher relative humidity, excess molecular water displaces chemisorbed O_2_^–^ species from oxygen-vacancy sites, suppressing the charge-transfer pathway required for room-temperature sensing. Moreover, excessive water vapor forms a physisorbed multilayer water film that acts as a physical diffusion barrier, blocking NO_2_ molecules from reaching the active surface sites, causing the response to drop.^5^ The strong dependence of the response of the sensor on RH indicates that humidity-control strategies, such as a PTFE hydrophobic membrane, would be required in practical applications. Further measurements at sub-ppm of NO_2_ concentrations under different RH levels would be needed to assess the sensor performance under real environmental conditions.

**Figure 8:**
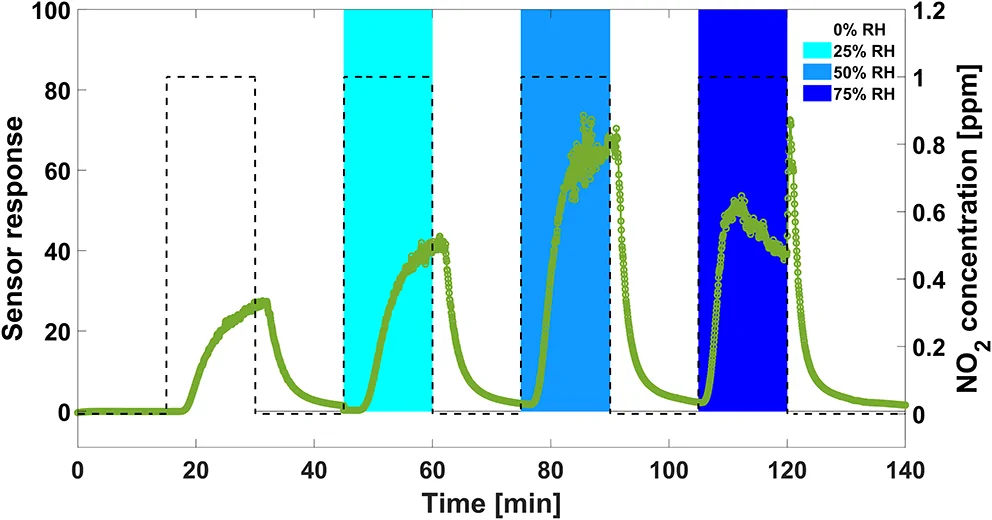
Sensor response of the ZnO-based gas sensor at different relative humidity (% RH) levels and 1 ppm of NO_2_.

### 3.2 Effect of Annealing

In order to investigate the influence of the annealing temperature on the gas sensing properties of ZnO films, we produced a set of sensors using three different drying strategies. Initially, all of the sensors were fabricated and dried for 24 h at room temperature. Following this, the sensors were divided into three groups:

1. Sensors were dried only at room temperature for 24 h.
2. Sensors were dried at room temperature for 24 h, then annealed in an oven at 110 °C for 10 min.
3. Sensors were dried at room temperature for 24 h, then annealed in an oven at 110 °C for 20 min.

Finally, we measured and compared the response of the sensors to NO_2_ gas (mixed with dry air), as shown in Figure 9.

**Figure 9:**
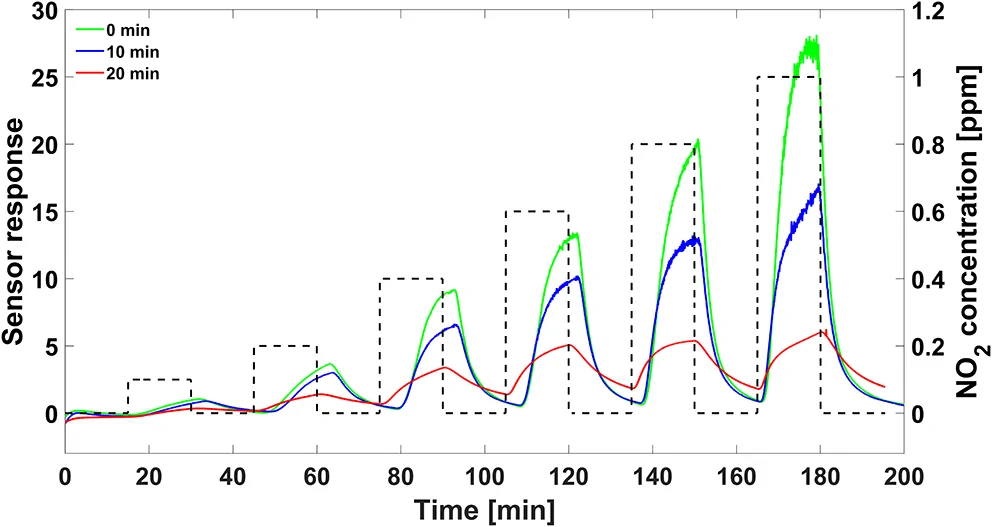
Sensor response of ZnO-based gas sensor for different annealing times at 110 °C.

The sensors annealed at 110 °C for 10 and 20 min demonstrate reduced sensitivity compared to those just cured at RT. Additionally, as the duration of the annealing process increases, the response of the sensors diminishes. As the annealing temperature is too low to produce a sensible grain size increase, the main cause of the decrease in sensitivity can be attributed to the modification of the packing properties of the film. In particular,^31^ reports greater nonuniformity of ZnO films when annealed at low temperatures.

### 3.3 Selectivity

As is well-known, selectivity to a specific gas is one of the main problems that most chemiresistive sensors face. In general, selectivity is the preferential sensitivity of a sensor for a specific gas in the presence of other gases under the same operating conditions. The selectivity can be expressed as

2 $$\mathrm{Selectivity}=\frac{S_{i}}{S_{g}}$$

where S_*i*_ is the sensor response toward a certain interfering gas and S_*g*_ is the sensor response toward the target gas.^5,32^

In the selection of the gases for the selectivity analysis, the application of the sensor and the principle of operation were considered. The most common air pollutant gases are carbon monoxide, sulfur dioxide, chlorofluorocarbons and nitrogen oxides produced by industry and vehicle combustion. Therefore, we tested most gases present in the atmosphere, such as oxygen (O_2_, 10^6^ ppm), carbon monoxide (CO, 50 ppm), carbon dioxide (CO_2_, 10^3^ ppm), methane (CH_4_, 50 ppm), sulfur dioxide (SO_2_, 10 ppm), and formaldehyde (CH_2_O, 25 ppm). In their original concentrations, all of these gases are mixed with air, except O_2_, which is pure, and formaldehyde, which is mixed with N_2_. To obtain the desired concentrations, all of the gases are mixed with synthetic air, except for O_2_, which is used in its original concentration. The selectivity test was performed using 0.1 ppm of NO_2_ and higher concentrations of potential interfering gases in order to evaluate the sensor response under a demanding cross-sensitivity scenario. In all of the experiments, the flow is maintained at 400 sccm.

The results presented in Figure 10 and Table 1 indicate a preferential response toward NO_2_ compared with most of the tested interferents. However, the non-negligible response observed for O_2_ suggests that oxygen-related baseline effects should be considered in practical operations. Further studies using interfering concentrations closer to typical ambient levels would be useful to fully assess the sensor selectivity under real environmental conditions.

**Figure 10:**
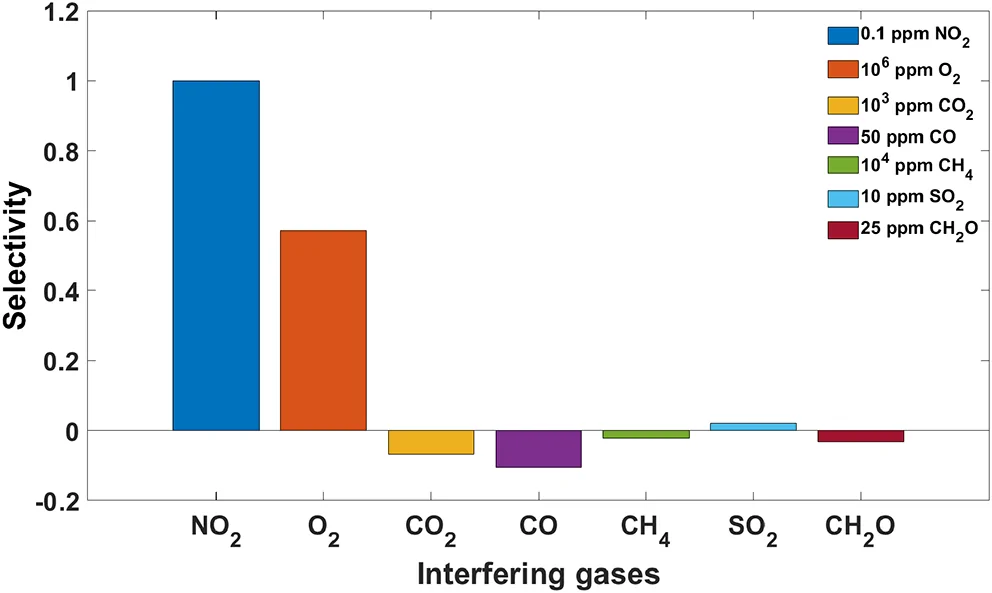
Selectivity of ZnO sensor to interferent gases under light stimulation at room temperature.

**Table 1:** Selectivity Values for Tested Interfering Gases

Interfering gas	Concentration (ppm)	Selectivity (Si/Sg)
NO_2_	0.1	1
O_2_	10^6^	0.57
CO_2_	10^3^	–0.067
CO	50	–0.105
CH_4_	10^4^	–0.022
SO_2_	10	0.021
CH_2_O	25	–0.033

In addition, the corresponding response of the ZnO film to reducing gases such as CO and CO_2_ is inverted due to the n-type semiconductor operating principle, causing an opposite effect.

### 3.4 Stability

The chemiresponsive material used in this study was unprotected from the atmosphere. Thus, its performance could degrade over time. Therefore, we checked its long-term stability in air to find its practical application capability. The response of the sensor after 6 months of operation is presented in Figure 11 (red line).

**Figure 11:**
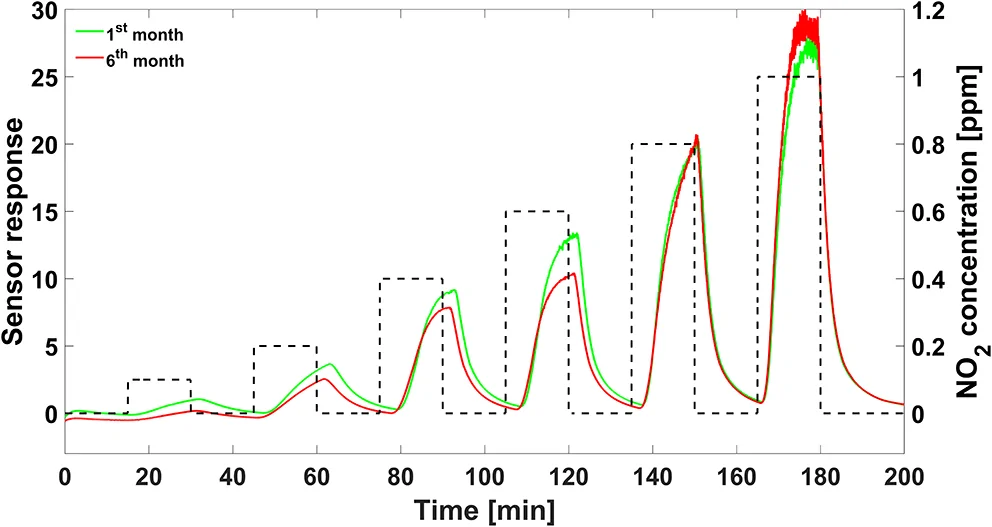
Response of a ZnO-based sensor after 6 months at room temperature.

As can be seen, the sensor could still accomplish effective detection with a fast response and full recovery under light-assisted conditions. Moreover, there was only a small decrease in the sensor response for concentrations below 0.6 ppm, demonstrating the stability of the ZnO film over time.

## 4 Discussion and Conclusions

A comparative analysis of NO_2_ sensing performance at room temperature, including the present sensor and previously tested ZnO-based sensors, is summarized in Table 2 and Table 3.

**Table 2:** Sensing Properties Comparison of Sensors for NO_2_ Detection

Sensitive materials	NO_2_ concentration (ppm)	Operating temperature (°C)	Sensor response	Reference
ZnO films	0.1 to 1	RT^*a*^ (light from fluorescent lamps)	1 at 0.1 ppm ((*R*_gas_–*R*_air_)/*R*_air_)	[this work]
ZnO nanofilms	0.025	RT^*a*^ (UV light, 365 nm, 25 μW/cm^2^)	1.65 at 25 ppb ((*R*_gas_–*R*_air_)/*R*_air_)	^15^
ZnO nanorod/SnO_2_ nanoparticles	0.5	RT^*a*^ (UV light, 380 nm, 0.7 cd/m^2^)	1266 at 0.5 ppm ((*R*_gas_–*R*_air_)/*R*_air_)	^18^
ZnO-Ag nanoparticles	0.5 to 5	RT^*a*^ (Visible light 470 nm, 75 mW/cm^2^)	1.545 at 1 ppm ((*R*_gas_–*R*_0_)/*R*_0_)	^9^
ZnO nanorod/Au hybrid	1	RT^*a*^ (Visible light, 532 nm, 2 mW/cm^2^)	3.66 at 1 ppm (*R_g_*/*R_a_*–1)	^8^
ZnO nanowires	1	RT^*a*^ (UV light, 360 nm, n/a)	7.08 at 1 ppm ((*R*_gas_–*R*_0_)/*R*_0_)	^14^
SnO_2_-core/ZnO-shell nanowires	1 to 5	RT^*a*^ (UV light 365 nm, 1.2 mW/cm^2^)	1.38 at 1 ppm (*R_g_*/*R_a_*–1)	^10^
ZnO mesoporous sheets	1	RT	1.35 at 1 ppm ((*R*_gas_–*R*_air_)/*R*_air_)	^28^

**Table 3:** Response and Recovery Time Comparison of Sensors for NO_2_ Detection^a^

		Response time (min)		Recovery time (min)		Reference
Sensitive materials	NO_2_ concentration (ppm)	dark	UV light	dark	UV light	
ZnO films	0.1	10	10	n/r	10	[this work]
ZnO nanofilms	0.025	n/a	n/a	n/a	n/a	^15^
ZnO nanorod/SnO_2_ nanoparticles	0.2	12	7	14	8	^18^
ZnO-Ag nanoparticles	1	n/a	2.4	n/a	2.2	^9^
ZnO nanorod/Au hybrid	1	n/a	n/a	n/a	n/a	^8^
ZnO nanowires	1	5	0.5	p/rv	2.4	^14^
SnO_2_-core/ZnO-shell nanowires	5	n/a	1.67	n/a	3.67	^10^
ZnO mesoporous sheets	1	3	-	2.5	-	^28^

a n/a: not available, n/r: no recover, and p/rv: poor reversibility.

Compared with the existing NO_2_ gas sensors, the developed ZnO-based gas sensor demonstrates high sensitivity, fast response and full recovery at room temperature. In particular, it is worth highlighting the performance of the sensor in the recovery stage under artificial light conditions without specific light or heat treatment that aids recovery.

Most of the ZnO-based gas sensors mentioned in the reviewed literature for NO_2_ detection use specific light stimulation from either the UV or visible spectrum, with the exception of the study by ref 28 and our own work (see ref Table 2). In ref 28, the sensor response for 1 ppm concentration of NO_2_ gas is reported as 1.35, which is higher than the sensor response obtained for the same concentration from our experiments. However, we also tested a concentration ten times lower. One notable advantage developed by ref 28 is its ability to operate without requiring light stimulation. This stands in contrast to the majority of other sensors that rely on specific light wavelengths for functionality. Nevertheless, the developed sensor in this work demonstrates the capability to detect concentrations down to 0.1 ppm of NO_2_. It is worth mentioning, however, that ref 15 achieved an even higher response at a lower concentration of 25 ppb, albeit under UV light.

It is worth mentioning that the longer response time of our ZnO film-based sensor compared to those in Table 3 is due to its dense, continuous structure. This morphology offers a lower surface-to-volume ratio and higher gas diffusion barriers than the nanoengineered morphologies presented in the referenced works, explaining both the greater response and recovery times registered.

In conclusion, we have developed a chemiresistive sensor that can operate at room temperature for air quality monitoring systems. This sensor was fabricated on a flexible, inexpensive, disposable substrate using a quick and easy thermal printing technique. The ZnO nanoparticle ink has proven to be a good room-temperature NO_2_ detection material, environmentally friendly, with easy availability and low cost. For the detection of NO_2_, high sensitivity, remarkable repeatability, rapid response, full recovery, strong selectivity and outstanding long-term stability were achieved.
